# Thermal Characterization of Medium-Temperature Phase Change Materials (PCMs) for Thermal Energy Storage Using the T-History Method

**DOI:** 10.3390/ma14237371

**Published:** 2021-12-01

**Authors:** Paulina Rolka, Roman Kwidzinski, Tomasz Przybylinski, Adam Tomaszewski

**Affiliations:** Institute of Fluid Flow Machinery, Polish Academy of Sciences, 80-231 Gdansk, Poland; rk@imp.gda.pl (R.K.); tprzybylinski@imp.gda.pl (T.P.); atomaszewski@imp.gda.pl (A.T.)

**Keywords:** phase change materials (PCMs), T-history method, thermal conductivity of PCMs, pipe Poensgen apparatus, latent heat thermal energy storage (LHTES)

## Abstract

To reduce energy consumption and increase energy efficiency in the building sector, thermal energy storage with phase change materials (PCMs) is used. The knowledge of the thermophysical properties and the characteristics of PCMs (like their enthalpy changes and the distribution of stored energy over a specified temperature range) is essential for proper selection of the PCM and optimal design of the latent thermal energy store (LHTES). This paper presents experimental tests of the thermophysical properties of three medium-temperature PCMs: OM65, OM55, RT55, which can be used in domestic hot water installations and heating systems. Self-made test chambers with temperature control using Peltier cells were used to perform measurements according to the T-history method. In this way the temperature range of the phase transition, latent heat, specific heat capacity, enthalpy and the distributions of stored energy of the three PCMs were determined. The paper also presents measurements of the thermal conductivity of these PCMs in liquid and solid state using a self-made pipe Poensgen apparatus. The presented experimental tests results are in good agreement with the manufacturers’ data and the results of other researchers obtained with the use of specialized instruments. The presented research results are intended to help designers in the selection of the right PCM for the future LHTES co-working with renewable energy systems, waste heat recovery systems and building heating systems.

## 1. Introduction

Energy consumption in the building sector accounts for 40% of global consumption and is responsible for 36% of greenhouse gas emissions to the atmosphere [1]. Just in Europe half of the total energy consumption is used for heating and cooling of residential and non-residential buildings, and about 84% of this energy is still generated from fossil fuels [2]. Today it is necessary to reduce the burning of fossil fuels and increase the use of renewable energy sources. Therefore, technologies integrating renewable energy sources with energy storage are required as well as actions aimed at the development of energy storage system technologies and their application.

Storage of heat or cold can be done with thermal energy storage (TES) using phase change materials (PCMs). PCMs incorporated in latent heat energy storages (LHTES) are used to store heat from solar collectors [3,4], to store energy from household wind power plants [5], in building heating and cooling systems [6,7], to support heat pumps and waste heat recovery installations [8], to support district heating networks [9,10]. In addition, PCMs are used to cool photovoltaic panels [11] and are also used in building structural elements like walls [12,13,14], windows [15] and roofs [16].

The appropriate use of PCMs and the design of LHTES in practical applications requires an in-depth knowledge of the thermal properties of PCMs. Basic thermal characteristics of PCMs are determined using thermal analysis techniques, of which differential scanning calorimetry (DSC) and the T-history method are the most widely used [17,18,19]. The DSC method allows one to determine the latent heat value, the melting/solidification temperature and the specific heat of the materials tested. Using the data measured by the DSC method, it is also possible to represent the enthalpy change versus temperature and determine the amount of stored/released energy in a given temperature interval [18]. However, the DSC method has some limitations like the small amount of sample to be tested (1–10 mg [20], 2–50 mg [21], less than 90 mg [17]), the effect of sample mass on the thermal response [2,17,18] and dependence of the results on the heating rate used [2,17,18]. Furthermore, repeatability may not be achievable for heterogeneous samples and in the case of composite materials (including composite PCMs), the main component may interfere with the PCM’s DSC signal [17]. The T-history method (THM), being an alternative to DSC measurements, allows one to determine the same thermophysical properties as DSC. The T-history method eliminates the previously mentioned limitations of DSC measurements (i.e., effect of mass size, influence of heating/cooling rate) and also allows for measurement of much larger samples than DSC (about 1000 times larger) [2]. Although the kind of calorimeter used during T-history measurements of PCMs can be made from simple laboratory equipment, it has a disadvantage that the accuracy of the measurement results is highly dependent on the measurement procedures implemented and the design of the calorimeter used for PCMs studies [2]. For this reason, measurements using the T-history method from different laboratories will not be identical and often may differ from DSC results. A comparison of the advantages and disadvantages of the DSC method and T-history method is presented in Table 1.

Specialized techniques are required to measure the thermal conductivity of PCMs. They usually involve transient or steady-state temperature measurements. Transient methods like transient hot wire/hot strip, transient plane source (TPS), laser flash analysis (LFA), and the thermocouple method are used to determinate the thermal conductivity of pure PCMs, microencapsulated PCMs, PCM composites (ceramic- and metallic-based), nano-enhaced PCMs, nanofluids and PCM slurry/suspensions/emulsions [22]. The most popular method to testing the thermal conductivity of pure PCMs and PCM composites are TPS, LFA and transient hot wire [23]. However, the determination of thermal conductivity using these methods requires the use of expensive and specialized equipment. Steady-state temperature measurements like guarded hot plate and heat flow meter are mainly used to determine the thermal conductivity of PCM composites and packed beds [23]. Among steady-state methods that are suitable for measurements in liquids and solids is the so-called pipe Poensgen apparatus. It is relatively simple measurement system which can be assembled using common laboratory equipment.

Published T-history method results are collected in Table 2 for the following selection of middle-temperature PCMs: C58 [19], OM55 [21], Na_2_S_2_O_3_·5H_2_O [24], mixture of 37.5 wt% NH_4_NO_3_ and 62.5 wt% of Mg(NO_3_)_2_·6H_2_O [24], myristic acid [24], lauric acid [24,25], mixture 58.7 wt% Mg(NO_3_)_2_·6H_2_O + 41.3 wt% MgCl_2_·6H_2_O [24], Mg(NO_3_)_2_·6H_2_O [24], sodium acetate hydrate CH_3_COONa·H_2_O [25] and trihydrate CH_3_COONa·3H_2_O (SAT) [20,24,26], Greek market paraffin [27], GR41 [28], RT45 [29], RT55 [29,30], RT58 [31,32], bischofite [32], D-mannitol [32] and hydroquinone [32].

A comparison of the thermo-physical properties of OM55 [21] and Greek market paraffins [27] obtained by T-history and DSC measurements shows that the phase transition temperatures of PCMs determined by both methods are almost identical, but the latent heat values may differ. For OM55 the latent heat values obtained by T-history and DSC differ by about 5 kJ/kg, while for Greek market paraffins the difference is larger and amounts to about 10–20 kJ/kg. On the other hand, tests performed on a large-scale LHTES storage with C58 material [19] show a smaller subcooling effect and a 10% lower amount of stored energy than indicated by the T-history measurement. Therefore, the authors of the study [19] emphasized that there is a need to compare the results of PCM characterization resulting from methods using different sample scales, such as DSC (milligram scale), T-history method (gram scale) and laboratory-size storage (kilogram scale). A similar comparison of the results obtained from the T-history and DSC methods, and the experimental studies of the large-scale energy storage LHTES can be found in [32] for the materials RT58, bischofite, D-mannitol and hydroquinone. The results of [32] presented in the form of enthalpy curves indicate the volume-independent melting and crystallization behavior of RT58, the occurrence of subcooling in bischofite, D-mannitol and hydroquinone (which are lower for measurements in pilot plant with LHTES storage than in T-history and DSC measurements), as well as good enthalpy curve agreement between the measurement methods. The authors of [32] suggest that comparisons of the results of enthalpy changes obtained by different methods and under different measurement conditions should be done using enthalpy–temperature diagrams.

The main objective of this paper is to determine the thermo-physical properties of several phase change materials (PCMs), namely OM65, OM55, RT55, with a medium temperature range of phase transition that can be applied for heat/cooling storage integrated with domestic hot water systems or district heating systems. This paper presents the experimental results of melting and solidification temperatures, latent heat, specific heat of OM65, OM55, RT55 and characteristic of enthalpy change and distribution of energy stored at a specific temperature range for these PCMs, obtained using the T-history method. Self-made test chambers with temperature control by Peltier cells were used to perform the measurements according to the T-history method. The paper also presents the construction of a self-built pipe Poensgen apparatus and its custom application to measure the thermal conductivity of PCMs in the liquid and solid state. The results of our experimental tests were compared with the results of other researchers and with manufacturers’ data which determined the thermo-physical properties of the PCMs using commercial devices like DSC equipment, KD2Pro and TPS-500 analyzers. The results presented here fully represent the characteristics of PCMs and provide the necessary knowledge for the proper design of LHTES storage with PCM and other uses of the investigated PCMs.

## 2. Materials, Methods and Experimental Set-Up

### 2.1. Materials

Experimental studies using the T-history method were performed for three commercially available organic PCMs with a medium temperature of phase transition: RT55 (Rubitherm, Berlin, Germany), OM55 (PLUSS, Gurugram, Haryana, India), OM65 (PLUSS, Gurugram, Haryana, India). The selected materials RT55 and OM55 have phase changes in the temperature range from 54 °C to 57 °C and could be used with domestic hot water systems. On the other hand OM65 has a phase change in the temperature range from 65 °C to 68 °C and could be used in district heating network systems. RT55 is a paraffin wax, while OM55 and OM65 are mixtures of fatty acids. Typical of this kind of materials, they show considerable volume expansion during their phase change and a low thermal conductivity.

### 2.2. T-History Method

The T-history method (THM) was developed by Zhang et al. in 1999 [24] and then refined by several researchers, including Hong et al. [25] and Marín et al. [34]. The T-history method is based on recording the temperature as a function of time for a PCM sample and a sample of reference material. Initially both samples are in equilibrium with the environment at constant temperature that is significantly higher or lower than the PCM phase transition temperature. The THM test starts when the PCM and reference samples are suddenly immersed into environment with a temperature different than the equilibrium temperature of samples. Thus, if the temperature of the PCM and the reference is below the phase change temperature, they should be placed in an environment with a temperature significantly higher than the phase change temperature to realize the phase transition during the heating process. In the other case, when the temperature of the samples is higher than the phase change temperature, they should be placed in an environment of sufficiently low temperature to realize the phase transition during a cooling process. For this purpose, the test samples are, for example, immersed in a water bath or put into a thermal chamber at a suitable temperature. The reference material for the tests in the temperature range from 0 to 100 degrees Celsius is usually water [35]. In the experiment, thermocouples are placed in the center of the samples with PCM and reference material, and another thermocouple is positioned at some distance from the samples. Registration of measurement data provides two temperature vs time curves, one for the PCM and the other for the reference, and a third for the environmental temperature. The areas under the PCM and reference temperature curves are compared with each other to determine the thermo-physical properties of the PCM. The application of the T-history method is justified when the Biot number meets the criterion Bi < 0.1 [24].

T-history method allows to determinate thermo-physical properties like temperature of phase change, latent heat and specific heat of PCMs.

In the original method of Zhang et al. [24], latent heat *L_t_* and specific heat *c_p_* at solid and liquid state could be obtained from the following formulas:

(1) $$c_{ps}=\left(\frac{m_{w}c_{pw}+m_{t}c_{pt}}{m_{p}}\right)\frac{I_{3}}{I_{2}^{\prime }}-\frac{m_{t}}{m_{p}}c_{pt}$$

(2) $$c_{pl}=\left(\frac{m_{w}c_{pw}+m_{t}c_{pt}}{m_{p}}\right)\frac{I_{1}}{I_{1}^{\prime }}-\frac{m_{t}}{m_{p}}c_{pt}$$

(3) $$L_{t}=\left(\frac{m_{w}c_{pw}+m_{t}c_{pt}}{m_{p}}\right)\frac{I_{2}}{I_{1}^{\prime }}\left(T_{0}-T_{s}\right)$$

If the PCM does not have a constant phase change temperature but rather the phase transition process takes place in a temperature range, to calculate the PCM latent heat *L_t_*, formula proposed by Marín et al. [34] could be used:

(4) $$L_{t}=\left(\frac{m_{w}c_{pw}+m_{t}c_{pt}}{m_{p}}\right)\frac{I_{2}}{I_{1}^{\prime }}\left(T_{0}-T_{s}\right)-\frac{m_{t\;}c_{pt}\;\left(T_{m1}-T_{m2}\right)}{m_{p}}\;$$

where *T_m1_*, *T_m2_* are the respective temperatures of the beginning and the end of the phase change transition.

Moreover, Marín et al. [34] proposed an extension of Equation (4) allowing one to determine the partial enthalpy Δ*h_p_* from the measured temperature history. Accordingly, the enthalpy increment Δ*h_p_* of the PCM over a small temperature interval Δ*T_i_* can be calculated as follows:

(5) $$\Delta h_{p}\left(T_{i}\right)=\left(\frac{m_{w}c_{pw}\left(T_{i}\right)+m_{t}c_{pt}\left(T_{i}\right)}{m_{p}}\right)\frac{I_{i}}{I_{i}^{\prime }}\Delta T_{i}-\frac{m_{t}}{m_{p}}c_{pt}\left(T_{i}\right)\Delta T_{i}$$

Then, the representation of h-T curve can be obtained as the integral of the partial enthalpy with respect to temperature, assuming some reference values of temperature and enthalpy. In the case of a PCM with a marked supercooling effect, the methodology presented by Hong [25] can be used to calculate the thermo-physical properties of the PCM.

### 2.3. Experimental Set-Up for T-History Method

**Figure 1:**
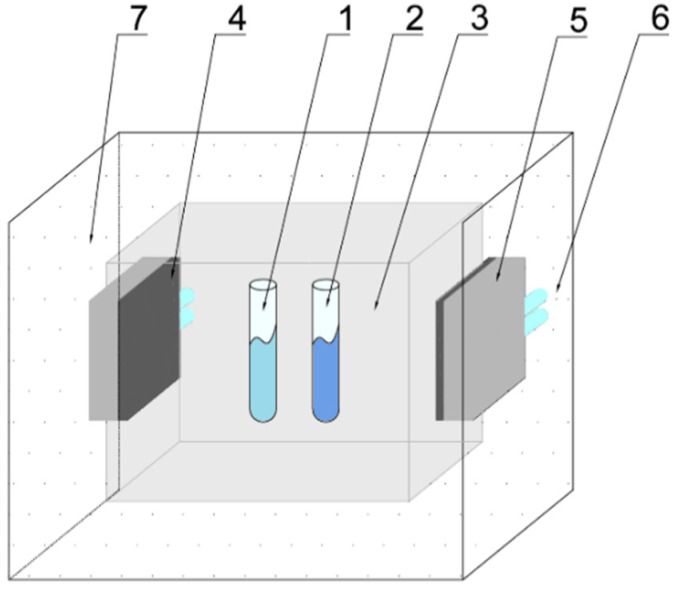
Schematic view of the own-designed thermal chamber: 1—test tube with PCM sample, 2—test tube with reference (water), 3—test chamber with air, 4—Peltier cell attached to the test chamber wall, 5—liquid heat sink, 6—connection to the cooling system, 7—thermal insulation.

**Figure 2:**
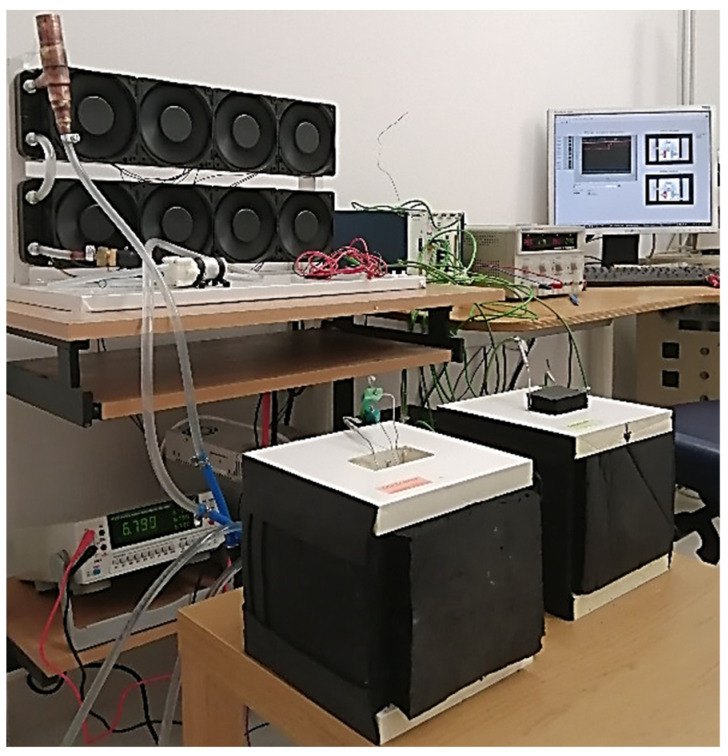
General view of the experimental set-up with own-design thermal chambers.

In order to determine the thermo-physical properties of PCMs using the T-history method, two thermal chambers were designed and built (see Figure 1 and Figure 2). The chambers allow for measurements in the temperature range from −8 to 80 °C. Each chamber is made of an aluminum (AMCO METALL-SERVICE GMBH, Brema, Germany) box that can be filled with air or water. Each wall of the aluminum box has dimensions 100 × 100 mm. The tested samples (PCM and reference) are first placed in one of the chambers to equalize their initial temperature and then in the other to realize the phase transition process. During the measurements, the PCM and reference fill test tubes with a diameter of 8 mm and a height of 100 mm. Peltier cells with a water heat sink (Thermonamic ElectronicsCorp. Ltd., Nanchang, China) were attached to three side walls of the box for cooling or heating of the test chamber. Setting the desired temperature inside the chamber is possible by appropriate control of the electric current and voltage supplied to the Peltier cells. To ensure adequate efficiency of Peltier cells they need to be cooled. Thus, one thermal chamber uses a liquid cooling system with an 8-fan cooler (AABCOOLING, Wieluń, Poland), mini circulation pump (FOTTON, Gliwice, Poland) and power supply unit (STAMOS, Germany). The cooling circuit of the other chamber is similar, but instead of the fan, a water cooler is used, which allows the temperatures in this chamber to drop below 0 degrees of Celsius. Both chambers are thermally insulated.

The test stand is equipped with a data measuring and acquisition system that allows one to record temperature changes over time. During the tests, the temperature inside both thermal chambers, the temperature of the PCM and the reference (the thermocouples positioned in the center of the test tubes), as well as the temperature in the laboratory are recorded. The accuracy of the temperature measurement is about ±0.2 K.

### 2.4. Thermal Conductivity of PCMs—Measurement Method and Device

To determine the thermal conductivity of the tested PCMs, it was decided to use a pipe Poensgen apparatus to perform the measurements of a steady-state temperature difference in a PCM sample of radial symmetry.

**Figure 3:**
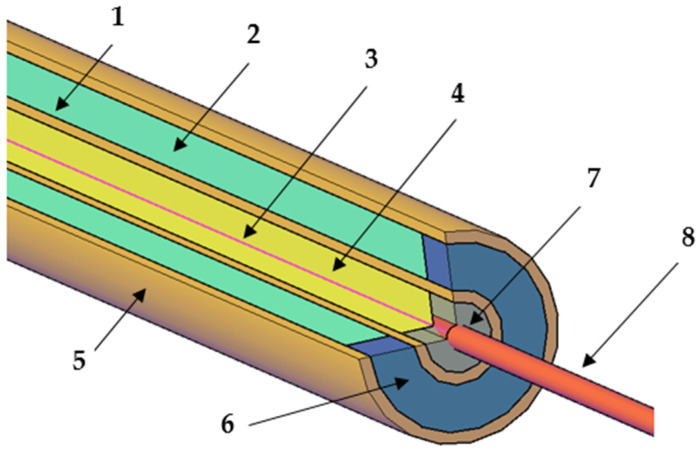
Schematic view of the self-made pipe Poensgen apparatus: 1—inner copper pipe, 2—layer of the tested material (PCM), 3—electric wire heater, 4—layer of sand, 5—outer copper pipe, 6—cap with insulating material, 7—cap, 8—insulated power connecting end of the electric heater.

**Figure 4:**
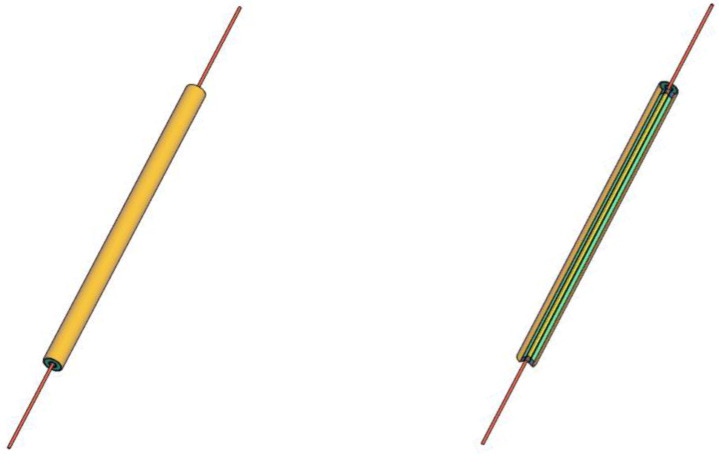
Schematic view of the self-made pipe Poensgen apparatus: external (**left**) and cross-sectional (**right**) view.

**Figure 5:**
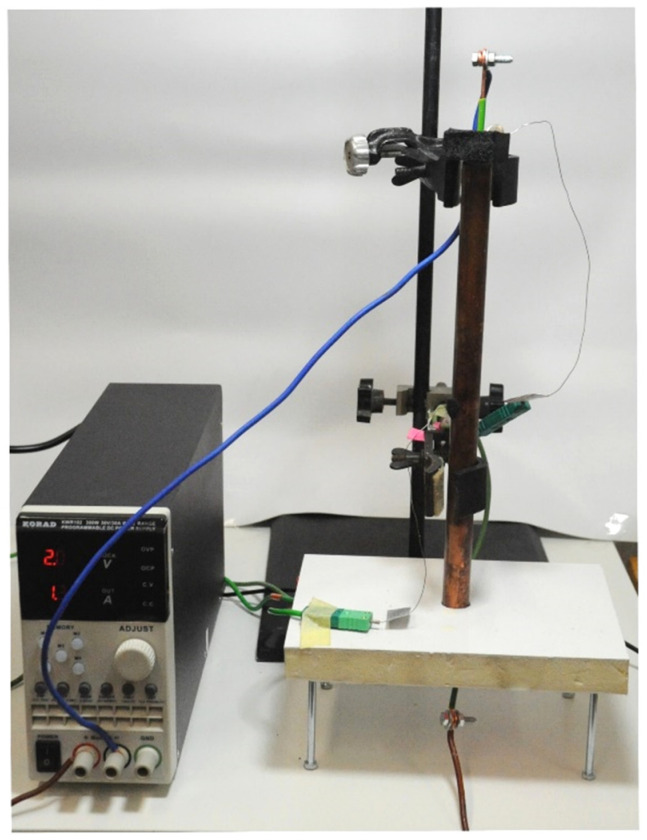
Self-made pipe Poensgen apparatus with power supply.

The pipe Poensgen apparatus (see Figure 3, Figure 4 and Figure 5) was also self–made. It consists of the following components: two copper pipes (Hutmen, Wrocław, Poland) an electric wire heater (ARPEM STEEL, Węgorzewo, Poland), an insulating material (Armacell, Środa Śląska, Poland), caps and thermocouples (CZAKI, Rybie, Poland). The electric heater (3) is placed centrally in the internal copper pipe (1) with an outer diameter of 8 mm. The space inside the inner copper pipe around the electric wire heater is filled with sand (4) to ensure even heat transfer without convection. The ends of the inner copper pipe (1) were closed with a cap (7) so that the power connections (8) to the electric heater (3) could protrude beyond the copper tube (1). A thermocouple was mounted at the outer wall of the inner copper tube (1) and the elements (1, 3, 4, 8) were placed centrally inside the outer copper pipe (5) with an inner diameter of 16 mm. Another thermocouple was mounted in the wall of the tube (5). The space between the two copper pipes (1 and 5) is filled with the tested PCM (2). The PCM sample is held in place with a special cap made of insulation material (6). The protruding ends of the electric heater were connected to the power supply and then insulated (8) in order to reduce heat losses to the environment.

The heat generated in the pipe Poensgen apparatus by the electric wire heater generates a steady temperature gradient in the tested sample. Thus the produced temperature difference in the sample in the vicinity of the two pipe walls is measured and used to calculate the thermal conductivity. The control of voltage and current in the electric wire heater means that the heat flow rate is known and can be controlled to achieve the desired temperature inside the Poensgen pipe apparatus. In this case, the total heat flow through the tested layer in steady-state conditions is determined from using Equation (6) [36].

(6) $$\dot{Q}=U\cdot I=\frac{2\pi \left(T_{w}-T_{z}\right)}{\frac{1}{\lambda }\cdot ln\frac{d_{z}}{d_{w}}}\cdot L$$

Transforming Equation (6), the thermal conductivity of tested material can be determined from:

(7) $$\lambda =\frac{U\cdot I\cdot ln\frac{d_{z}}{d_{w}}}{2\pi \cdot L\cdot \left(T_{w}-T_{z}\right)}.$$

### 2.5. Measurement Uncertainty

The uncertainty of the final results depends on individual uncertainties of the measuring instruments used during the experiments.

The absolute error of the T-type thermocouple was 0.2 °C, therefore the uncertainties of melting and solidification temperature of PCMs are ±0.2 °C.

The measurement uncertainty of the latent heat determined on the basis of Equation (8) [24] is about ±6% for OM55 and RT55 and for OM65 it is about ±12%. On the other hand, the specific heat measurement uncertainty determined according to equations (9) and (10) [24] is about 2–6%:

(8) $$\frac{\Delta L_{t}}{L_{t}}\approx \frac{4\Delta T}{T_{m}-T_{a}}+\frac{2\Delta T}{T_{0}-T_{m}}$$

(9) $$\frac{\Delta cp_{s}}{cp_{s}}\approx \frac{2\Delta T}{T_{m}-T_{a}}+\frac{2\Delta T}{T_{r}-T_{m}}$$

(10) $$\frac{\Delta cp_{l}}{cp_{l}}\leq \frac{4\Delta T}{T_{m}-T_{a}}$$

The total uncertainty of the thermal conductivity using experimental set-up of the Poensgen pipe apparatus is based on Equation (7) and was calculated from Equation (11) [37].

(11) $$\Delta \lambda =\sqrt{\overset{7}{\underset{i=1}{\sum }}{\left(\frac{\partial \lambda \left(X\right)}{\partial X_{i}}\Delta X_{i}\right)}^{2}},\;\;X=\left(U,I,L,d_{z},d_{w},T_{z},T_{w}\right)$$

As a result, the total uncertainty of the thermal conductivity is evaluated to be about 4% for liquid state and about 8–12% for solid state.

## 3. Results

Experimental tests using the T-history method for PCMs were carried out for samples of OM65, OM55 and RT55. Each sample was tested during their heating and cooling process and each measurement was repeated three times. The masses of the tested PCMs samples were: 2.05 g of RT55, 1.1 g of OM55, and 1.26 g of OM65, respectively. The thermal conductivity in solid and liquid states of these three PCMs was measured by the pipe method. The pipe Poensgen apparatus was filled with 46 mL of PCM in liquid state for each test, which corresponded to about 36 g RT55, 43 g OM65 and 39 g OM55 (see Table 3).

Based on the measured profiles of the temperature variation over time of the PCMs and the reference (water), from the equations of T-history method (presented in Section 2 this paper) the melting and solidification temperature, latent heat, specific heat, enthalpy distribution and heat capacity (energy stored) in the specified temperature range were obtained. The results of the experimental data analysis are presented below in Figures 6–23.

### 3.1. OM65

**Figure 6:**
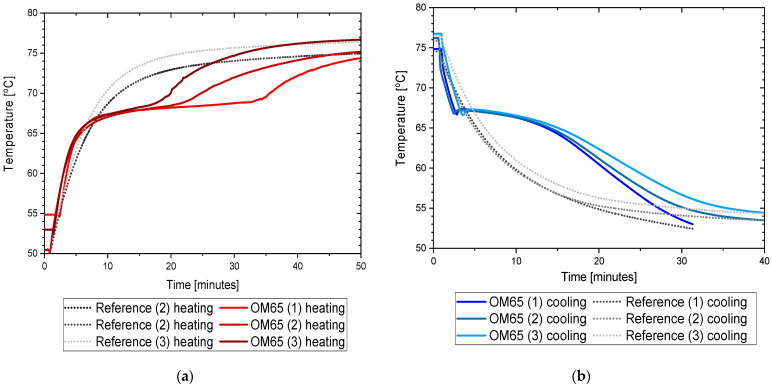
Temperature curve of PCM (OM65) and reference sample (water) during test: (**a**) heating process; (**b**) cooling process.

**Figure 7:**
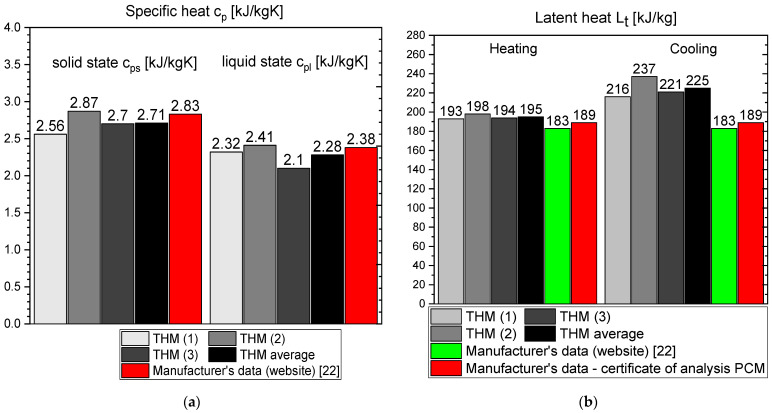
(**a**) Specific heat capacity of OM65 in solid (at 62 °C) and liquid (at 69 °C) state; (**b**) latent heat of OM65 [22].

**Figure 8:**
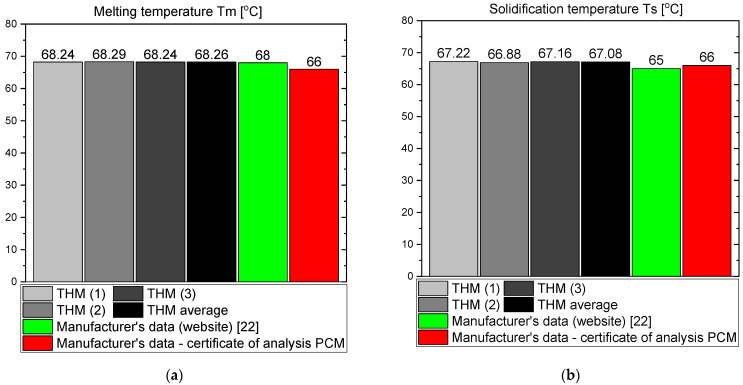
(**a**) Melting temperature of OM65; (**b**) solidification temperature of OM65 [22].

**Figure 9:**
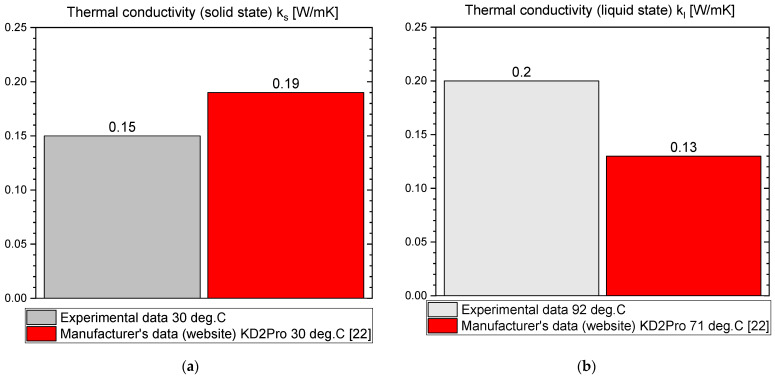
Thermal conductivity of OM65 in (**a**) solid state and (**b**) liquid state [22].

**Figure 10:**
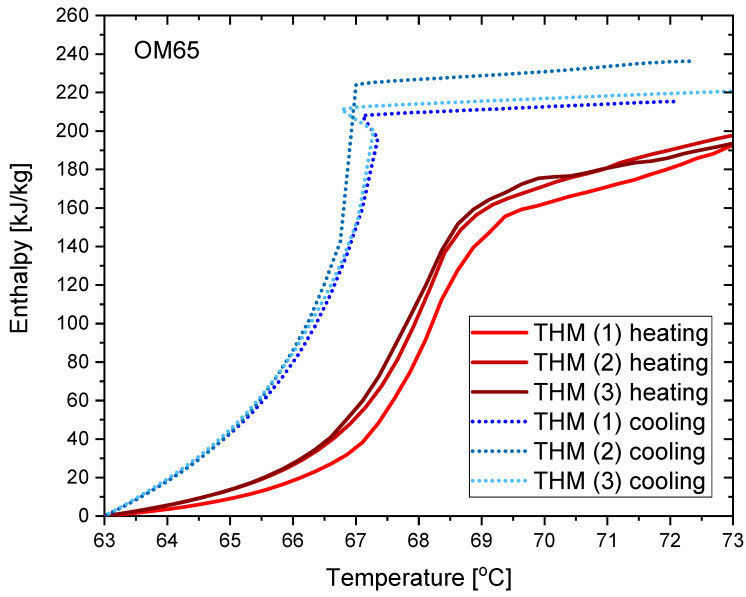
Enthalpy change of OM65 during heating and cooling process.

**Figure 11:**
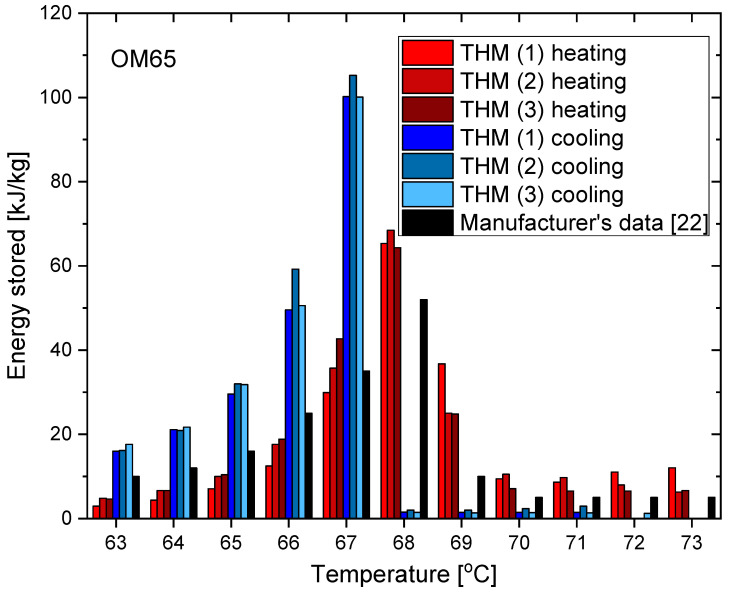
Distribution of energy stored during phase change of OM65 in heating and cooling process [22].

The results of the measurements for OM65 are presented below. The temperature curves of OM65 and reference (water) during the heating and cooling process are presented in Figure 6. The results of the specific heat are shown in Figure 7a and the latent heat in Figure 7b. The melting point results are presented in Figure 8a and the solidification point in Figure 8b. The results of thermal conductivity measurements in the pipe Poensgen apparatus with the PCM in solid and liquid states are presented in Figure 9. Moreover, Figure 10 shows the enthalpy curves for OM65, while Figure 11 presents distributions of energy stored.

### 3.2. OM55

**Figure 12:**
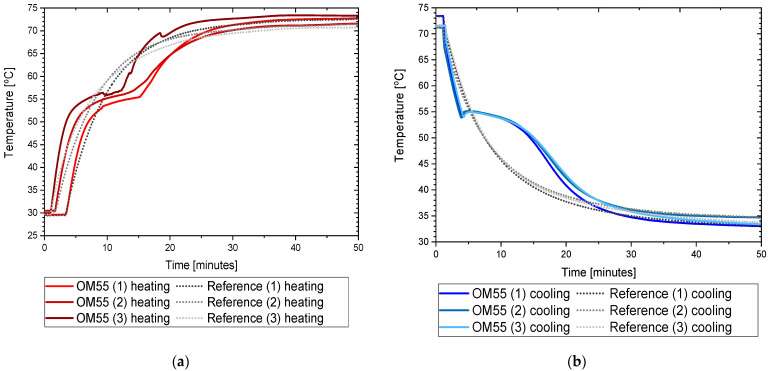
Temperature curve of PCM (OM55) and reference sample (water) during test: (**a**) heating process; (**b**) cooling process.

**Figure 13:**
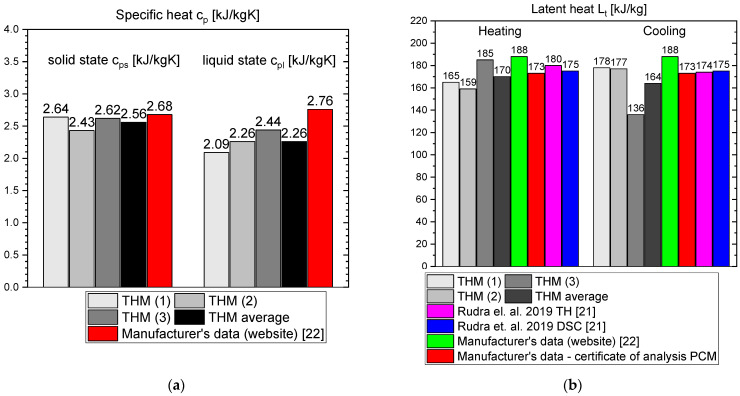
(**a**) Specific heat capacity of OM55 in solid (at 45 °C) and liquid (at 56 °C) state; (**b**) latent heat of OM55 [21,22].

**Figure 14:**
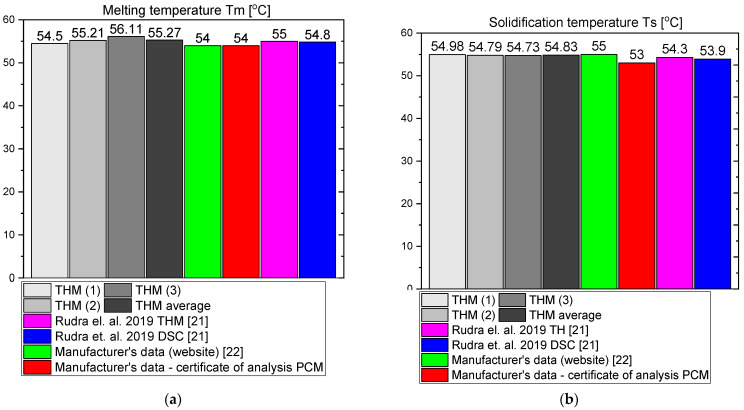
(**a**) Melting temperature of OM55; (**b**) solidification temperature of OM55 [21,22].

**Figure 15:**
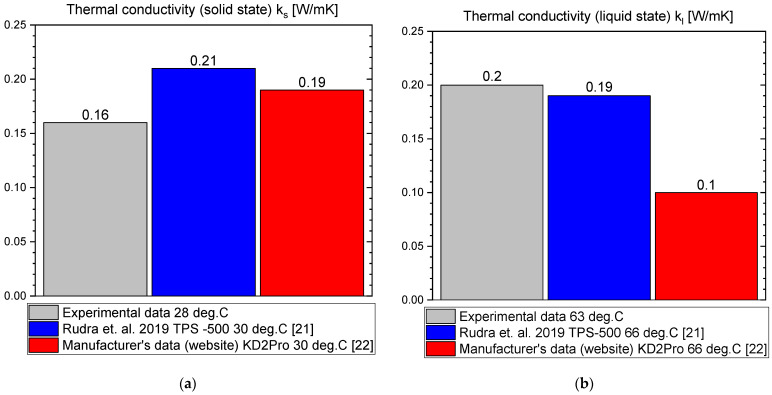
Thermal conductivity of OM55 in (**a**) solid state and (**b**) liquid state [21,22].

**Figure 16:**
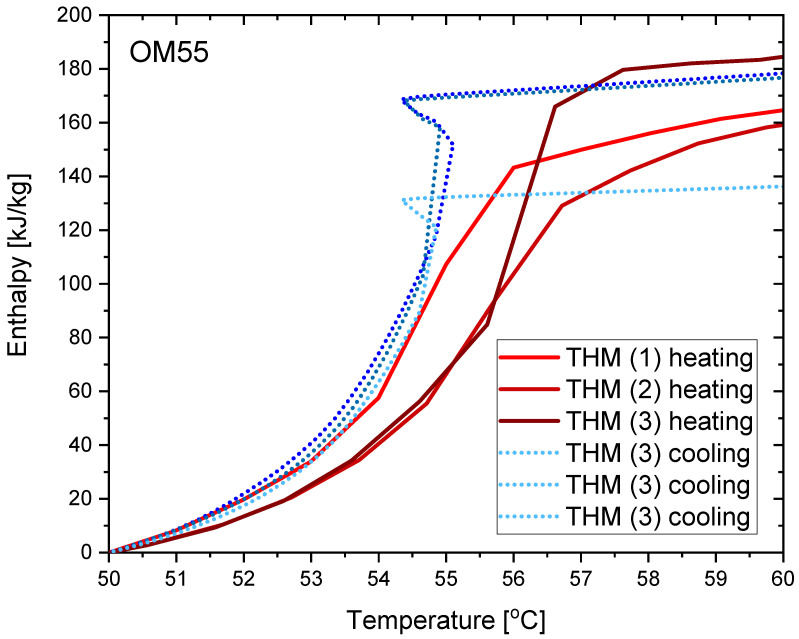
Enthalpy change of OM55 during heating and cooling process.

**Figure 17:**
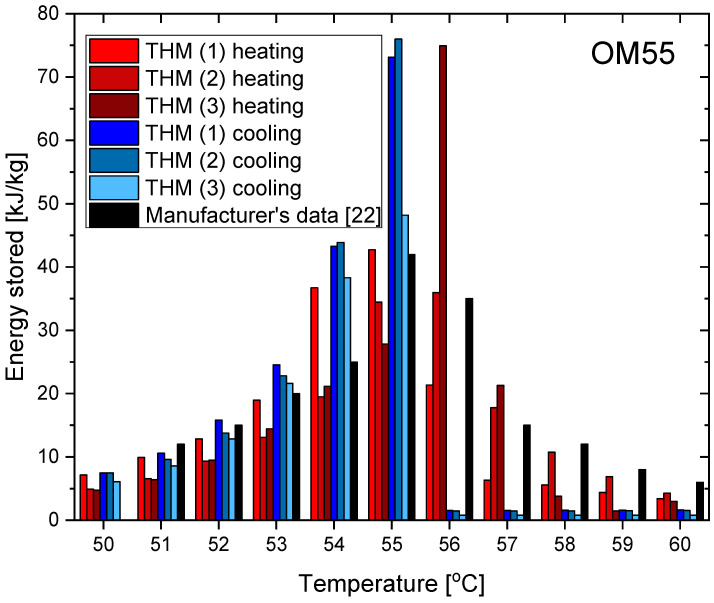
Distributions of energy stored during phase change of OM55 in heating and cooling process [22].

The measurement results for OM55 are presented in the Figures below. The temperature curves of OM55 and reference water sample during the heating and cooling process are presented in Figure 12. The results of the specific heat of OM55 are shown in Figure 13a, and the latent heat in Figure 13b. The melting point results are presented in Figure 14a and the solidification point in Figure 14b. The thermal conductivity measurements in the pipe Poensgen apparatus are presented in Figure 15 for the PCM in the solid and liquid states. Moreover, Figure 16 shows the enthalpy curves of OM55, while Figure 17 presents distributions of energy stored. The calculation results were also compared with the published results of other researchers (Rudra et al. [21]) and with the manufacturer’s data [22].

### 3.3. RT55

**Figure 18:**
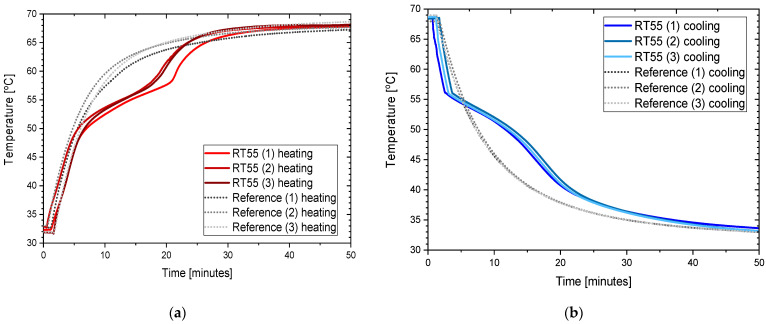
Temperature curve of PCM (RT55) and reference sample (water) during test: (**a**) heating process; (**b**) cooling process.

**Figure 19:**
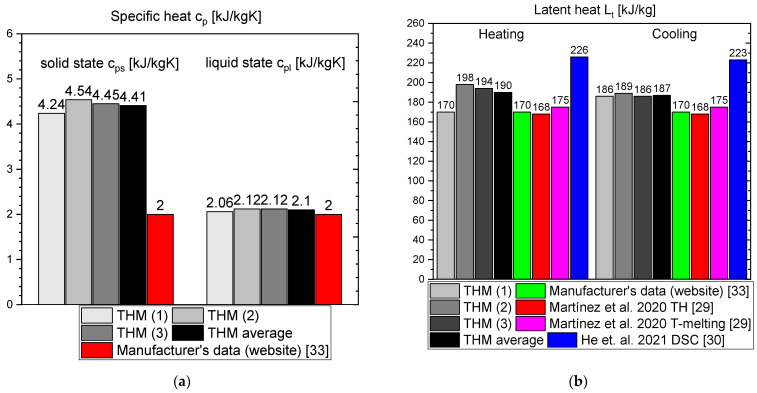
(**a**) Specific heat capacity of RT55 in solid (at 48 °C) and liquid (at 58 °C) state; (**b**) latent heat of RT55 [29,30,33].

**Figure 20:**
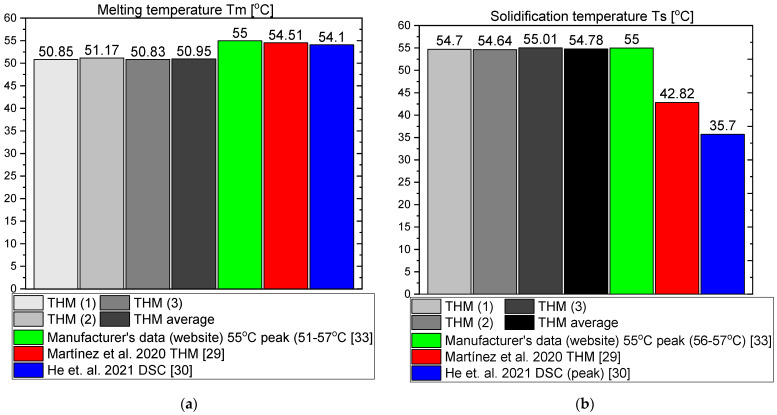
(**a**) Melting temperature of RT55; (**b**) solidification temperature of RT55 [29,30,33].

**Figure 21:**
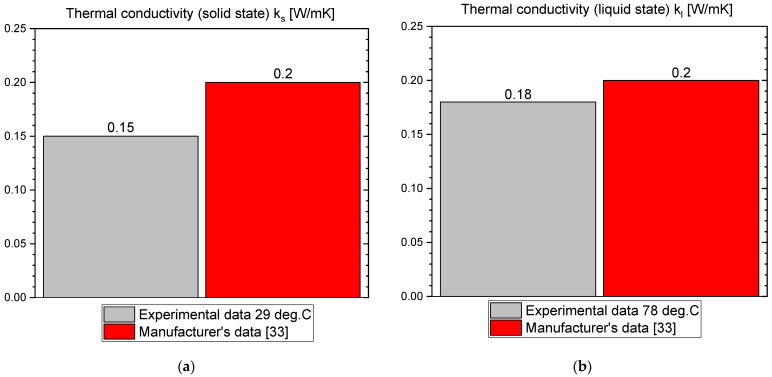
Thermal conductivity of RT55 in (**a**) solid state and (**b**) liquid state [33].

**Figure 22:**
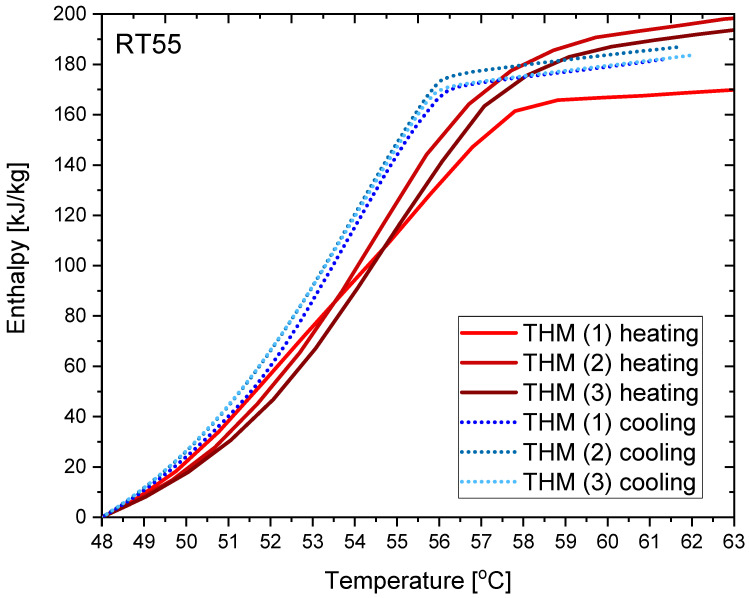
Enthalpy change of RT55 during heating and cooling process.

**Figure 23:**
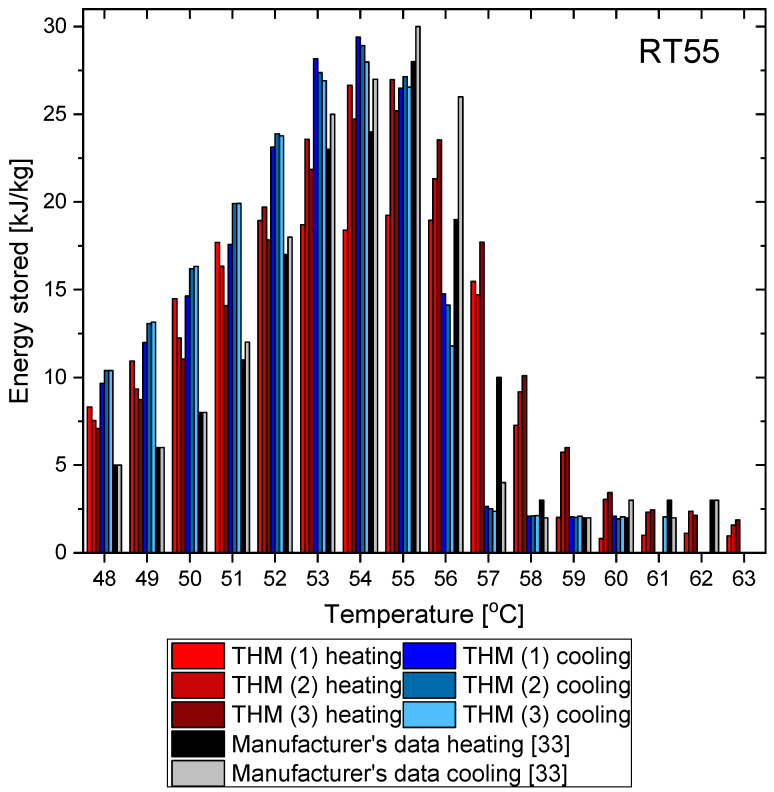
Distributions of energy stored during phase change of RT55 in heating and cooling process [33].

This section presents the results of the experimental research for RT55. The temperature curves of RT55 and the reference (water) during the heating and cooling process are presented in Figure 18. The specific heat of RT55 is shown in Figure 19a, and the latent heat in Figure 19b. The melting and solidification point values are presented in Figure 20a and Figure 20b, respectively. The PCM thermal conductivity in the solid and liquid states, measured in the pipe Poensgen apparatus is presented in Figure 21. Moreover, Figure 22 shows the enthalpy curves, while Figure 23 presents distributions of energy stored. These results are also compared with the published results of other researchers (Martinez et al. [29] and He et al. [30]) and with the manufacturer’s data [30].

## 4. Discussion

During the experiments, the OM65 was tested using the T-history method in the self-constructed calorimeter, in the temperature range from approximately 50 °C to 78 °C (see Figure 6). The value of the specific heat determined in the present experiment for the liquid state at about 69 °C was in the range of 2.1–2.41 kJ/kgK, and for the solid state at about 62 °C in the range of 2.56–2.87 kJ/kgK (Figure 7a). These values for the specific heat are similar to the manufacturer’s values 0f 2.38 kJ/kgK in the liquid and 2.83 kJ/kgK in the solid state [22]. The value of the latent heat obtained in the experiment (see Figure 7b) for the heating process (approx. 182 kJ/kg) and for the cooling process (approx. 186 kJ/kg) is consistent with the manufacturer’s values of 183–189 kJ/kg [22] and slightly lower than that given by Patil et al. [38] (210 kJ/kg). The melting point of OM65 during the conducted experimental research was approximately 66 °C (see Figure 8a), while the manufacturer reports a value of 68 °C (website) and 66 °C (PCM analysis certificate). On the other hand, the solidification temperature of OM65 during the tests was about 67 °C (Figure 8b), while the manufacturer specifies 65 °C (website) or 66 °C (certificate of analysis). In the work by Patil et al. [38], it is determined that phase transitions takes place in the range of 66–68 °C. The thermal conductivity of OM65 obtained from the pipe apparatus for the liquid state is approximately 0.15 W/m·K and approximately 0.2 W/m·K for the solid state (Figure 9). However, the thermal conductivity measured by the manufacturer using the KD2Pro analyzer is slightly lower and amounts to 0.13 W/m·K for the liquid state and 0.19 W/m·K for the solid state. The measurements of OM65 in the present experiment show good agreement with the manufacturer’s data, who tested a 20 g sample of OM65 and determined the melting point, solidification temperature and latent heat also using the T-history method.

OM55 tests conducted in the temperature range from 30 °C to 70 °C (see Figure 12) also showed good agreement with the manufacturer’s values. The value of the specific heat in the experiment was about 2.09–2.44 kJ/kgK for the liquid state at 56 °C and about 2.43–2.64 kJ/kgK for the solid state at 45 °C (Figure 13a). That is consistent with Rudra et al. [21] (2.3–3.1 kJ/kgK) and the data of the manufacturer [22] (2.68–2.76 kJ/kgK). The value of OM55 latent heat calculated from the experimental data is approximately 176 kJ/kg for the heating process and approximately 181 kJ/kg for the cooling process (Figure 13b). This is consistent with the manufacturer’s data (173–188 kJ/kg) and Rudra et al. (174–180 kJ/kg) [21].The melting point in the present experiment was about 54 °C (see Figure 14a), which corresponds to the value of 54 °C given by the manufacturer (T-history method) and approximately 55 °C provided by Rudra et al. [21] (T-history and DSC methods). The solidification temperature in the present test was approxmately 55 °C (Figure 14b), while the value given by the manufacturer is 53–55 °C (T-history method) and Rudra et al. [21] report a value of approximately 54 °C (T-history and DSC method). The thermal conductivity obtained from the present experiment was about 0.20 W/m·K for the liquid and about 0.16 W/m·K for the solid state (Figure 15). This parameter was also measured by Rudra et al. [21] using the hot disk method (TPS-500 analyzer) and the values of 0.19 W/m·K for the liquid and 0.21 W/m·K for the solid are reported. On the other hand, the thermal conductivity values obtained by the manufacturer with the KD2Pro device are 0.16 W/m·K for the solid state and 0.1 W/m·K for the liquid one [22]. Thus, the values of the OM55 thermal conductivity obtained from the three different measurement methods are generally similar.

In the experimental tests of RT55 (see Figure 18), the value of the specific heat for the liquid at 58 °C was approximately 2.06–2.12 kJ/kgK (see Figure 19a) and is similar to the value given by Martínez et al.—2.43 kJ/kgK [29], and comparable to the manufacturer’s value of 2 kJ/kgK. On the other hand, the value of specific heat for the solid form at 48 °C is approx. 4.24–4.54 kJ/kgK (Figure 19a) which is higher than manufacturer’s value of 2 kJ/kgK. In their research, Martínez et al. [29] give a value of 5.36 kJ/kgK for the solid state, which is even higher than the values obtained in this study and by the manufacturer. The value of the latent heat obtained in this work amounts to approximately 177 kJ/kgK for the heating and 178 kJ/kgK for the cooling process (Figure 19b), in good agreement with both Martínez et al. [29] and the manufacturer’s data [33], specified as 168.30 ± 3.29 kJ/kgK and 170 kJ/kgK, respectively. However the results of latent heat obtained by He et al. [30] in the DSC measurement are higher (226.2 kJ/kg for heating and 223.3 kJ/kg for cooling). The melting point was determined at about 55 °C (see Figure 20a), which is similar to value of 54 °C obtained by Martínez et al. [29], also with T-history method, and the value of 54.1 °C obtained by He et al. [30] by a DSC test. It is also within the manufacturer’s value range of 51–57 °C (with peak at 55 °C). In turn, the solidification point in the present experiment was approximately 55 °C (Figure 20b) that is in line with the range given by the manufacturer (56–57 °C, peak 55 °C) but significantly higher than the solidification temperatures of approximately 43 °C reported by Martínez et al. [29] and 35.7 °C reported by He et al. [30]. The thermal conductivities obtained in the present experiment are also similar to the values given by the manufacturer (0.2 W/m·K) and measured by Martínez et al. [29] using the T-melting method (approx. 0.2 W/m·K). Namely, in the present tests, the thermal conductivity for the liquid is about 0.18 W/m·K and about 0.15 W/mK for the solid (Figure 21). 

## 5. Conclusions

The paper presents the results of experimental determination of the thermo-physical properties for selected phase change materials (PCMs) that are obtained with the use of two self-built devices: a calorimeter (using our own thermal chambers) for T-history method measurements and a pipe Poensgen apparatus to measure thermal conductivity. Three commercially available PCMs—OM65, OM55 and RT55—were selected for the tests. These PCMs can be a part of thermal energy stores that, for example, can be used to support the operation of a heating network or the system for domestic hot water supply.

The results from the T-history measurements using the self-built calorimeter for the abovementioned three PCMs showed that:

- The phase transition takes place over the temperature range of 66–67 °C for OM65, 54–55 °C for OM55 and in approximately 55 °C for RT55, and these values are similar to results provided by the respective manufacturers and other researchers who used different methods of measurement (and often expensive instruments).
- The values of latent heat in the heating/cooling process obtained with the use of our calorimeter are 182/186 kJ/kg for OM65, 176/181 kJ/kg for OM55 and 177/178 kJ/kg for OM55, and are consistent with the values reported by the manufacturers and other researchers.
- The determined enthalpy–temperature curves reveal that the smallest hysteresis between melting and solidification corresponds to RT55 and it equals about 1 K (see Figure 22), while the hysteresis in OM65 and OM55 is about 1.5–2.0 K (see Figure 10 and Figure 16).
- Differences between different measurement methods were observed for the values of a single-phase specific heat. However, they are generally limited to the range of 0.12–0.6 kJ/kgK, except for RT55 that shows differences up to 2.5 kJ/kgK for the solid state.
- The distribution of partial enthalpy (stored energy) in the case of RT55 is spread over a temperature range of about 10–11 K and the energy stored or released over a 1 K temperature interval ranges from 7 to 30 kJ/kg (see Figure 23). On the another hand, the partial enthalpy distribution of OM55 and OM65 (see Figure 11 and Figure 17) is more concentrated in a narrower temperature range of 6–7 K and most of the energy is stored or released at the phase change peak (about 80 kJ/kg for OM55 and 110 kJ/kg for OM65).

The measurements of PCMs thermal conductivity with the pipe apparatus lead to the conclusions that thermal conductivity of:

- OM65 is 0.15 W/m·K in solid state and 0.19 W/m·K in liquid.
- OM55 is 0.16 W/m·K in solid state and 0.20 W/m·K in liquid.
- RT55 is 0.15 W/m·K in solid state and 0.18 W/m·K in liquid.

The thermal conductivity value of OM55 measured with the self-built apparatus is similar to the results obtained using commercial instruments like KD2Pro and TPS-500 analyzers.

The presented experimental research are intended to provide the necessary data for the correct design of a thermal energy storage system. The devices presented in the paper can be used to determine the thermo-physical properties of PCMs, but every effort should be made to ensure that the experimental set-up has the lowest possible measurement uncertainty.

Nevertheless, the research results regarding specific thermo-physical properties presented in the paper could be further developed in the future by:

- Comparison of the values of specific heat, latent heat and phase transition temperatures from the T-history method with the results from large-scale thermal energy stores (in which the mass of PCMs is several or several dozen kilograms) in order to determine the scale effect.
- Comparison of the PCMs thermal conductivity measurements at the same conditions using different measurement methods to better evaluate the measurement uncertainties.
- Carrying out research works aimed at increasing the thermal conductivity of PCMs by adding, for example, metal nanoparticles and determining their influence on the thermo-physical properties of PCMs.

## Figures and Tables

**Table 1:** Advantages and disadvantages of DSC method and T-history method [2,17,18,19,20,21].

Method	Advantages	Disadvantages
DSC method	short measurement time, heating/cooling rate control, the ability to test samples in a wide range of temperatures	small test sample (typically a dozen milligrams), effect of sample mass and heating/cooling rate on thermal response, expensive equipment
T-history method	simple and cheap laboratory equipment, relatively large test samples (typically dozens of grams)	long measurement time, substantial effort required to ensure accurate measurement results

**Table 2:** Summary of thermo-physical properties of middle-temperature PCMs tested experimentally using the T-history method: T-history and DSC results, and manufacturers’ data.

PCM Name	Manufacturer	T_m_ [°C]	T_s_ [°C]	L_sl_ [kJ/kg]	C_ps_ [kJ/kgK]	C_pl_ [kJ/kgK]	k_s_ [W/mK]	k_l_ [W/mK]	THM	DSC	M	Ref.
RT45	Rubitherm	44.33	35.95	166.72	4.35	2.22		0.23	X			[29]
RT45	Rubitherm	46	40	160	2	2	0.2	0.2			X	[29]
Na_2_S_2_O_3_·5H_2_O	-	48.0	-	206	-	3.83	-	-	X			[24]
GR41	Rubitherm	31–45	31–45	65.9	2.784	2.745	-	-	X			[28]
GR41	Rubitherm	33–48	33–48	64	-	-	-	-			X	[28]
37.5 wt% NH_4_NO_3_+62:5wt% Mg(NO_3_)_2_·6H_2_O	-	48.7	-	118	2.60	2.56	0.34	-	X			[24]
Myristic acid	-	49.7–52.7	-	201	2.91	3.67	0.17	-	X			[24]
Lauric acid	-	52.5	-	160		1.75	0.17	-	X			[24]
Lauric acid	-	-		186 ± 10	2.81 ± 0.60	2.14 ± 0.46	-	-	X			[25]
Hexacosane	-	54.60–57.69	-	216	3.89	2.94	0.22	-	X			[24]
Greek market paraffin	-	58 56	50 51	136.5 ± 6126.9 ± 6.5	-	-	-	-	X			[27]
Greek market paraffin	-			146.5						X		[27]
CH_3_COONa·3H_2_O	-	58.0	-	248	2.26	3.33	-	-				[24]
sodium acetate CH_3_COONa·H_2_O	-	-	-	245 ± 9	2.26 ± 0.13	3.74 ± 0.59	-	-				[25]
Sodium acetate trihydrate, CH_3_COONa·3H_2_O (SAT)	-	40–55	-	243	3.13	3.28	-	-				[26]
SAT	-	49.8–54.6	49.8–54.6	253.7	3.23	3.23	0.6349	0.6349	X			[20]
SAT	-	48.3–53.5	48.3–53.5	252.1	-	-	0.6326	0.6326		X		[20]
OM55	PLUSS	55	54.3	180–209	2.3	2.6	-	-	X			[21]
OM55	PLUSS	54.8	53.9	175–201	2.7	3.1	0.21	0.19		X		[21]
RT55	Rubitherm	54.51	42.82	168.30	5.36	2.43		0.2	X			[29]
RT55	Rubitherm	54.1	35.7	223–226	-	-	-	-		X		[30]
RT55	Rubitherm	57	56	170	2	2	0.2	0.2			X	[29]
C58	Climsel	57–61	55–50	216 ± 13	-	-	-	-	X			[19]
C58	Climsel	58	55	260	-	-	0.57	0.47			X	[19]
58.7 wt% Mg(NO_3_)_2_ ·6H_2_O +41:3 wt% MgCl_2_·6H_2_O	-	58.3		120	1.94	2.57	0.61	-				[24]
RT58	Rubitherm	-	-	176	1.9	2.6	-	-	X			[31]
RT58	Rubitherm	-	-	178	-	-	-	-			X	[33]
RT58	Rubitherm	52–62	52–62	162–167	-	-	-	-		X		[32]
RT58	Rubitherm	52–62	52–62	162–167	-	-	-	-		X		[32]
RT58	Rubitherm	52–62	52–62	167–170	-	-	-	-	X			[32]
Mg(NO_3_)_2_·6H2O	-	89.0	-	175	1.92	2.39	-	-				[24]
Bischofite	-	75–115	75–115	227–228	-	-	-	-		X		[32]
Bischofite	-	90–115	90–115	183–186	-	-	-	-	X			[32]
d-mannitol	-	115–165	115–165	396–400	-	-	-	-		X		[32]
d-mannitol	-	131–171	115–165	375–389	-	-	-	-	X			[32]
Hydroquinone	-	150–180	150–180	307–333	-	-	-	-		X		[32]
Hydroquinone	-	160–180	160–180	278–292	-	-	-	-	X			[32]

T_m_, T_s_—melting/solidification temperature (value or range); L_t_—latent heat; Cp_l_, Cp_s_—specific heat capacity of liquid/solid phase; k_l_, k_s—_thermal conductivity in liquid/solid phase; THM—measurement by T-history method; DSC—measurement by DSC; M —manufacturer data.

**Table 3:** Amount of PCMs tested in the Poensgen pipe apparatus.

PCM Name	Manufacturer	Volume of PCM Applied [mL]	Mass of PCM [g]	Density of PCM [kg/m^3^]	Ref.
OM65	PLUSS	46.0	38.7	924	[22]
OM55	PLUSS	46.0	42.5	841	[22]
RT55	Rubitherm	46.0	35.4	770	[33]

## Data Availability

Data is contained within the article.

## Nomenclature

*c_p_*	specific heat
*Δh_p_*	partial enthalpy
*d_w_*	inner pipe outer diameter
*d_z_*	outer pipe inner diameter
*I*	current, Equations (6), (7) and (11)
*I*	corresponding area under the PCM temperature curve, Equations (1)–(3)
*I’*	corresponding area under the reference temperature curve
*m*	mass
*λ*	thermal conductivity
*L*	pipe length
*L_t_*	latent heat
*PCM*	Phase Change Material
$\dot{Q}$	heat flow rate
*T*	temperature
*T_m_*	melting temperature of PCM
*T_w_*	temperature of the outer wall of the inner pipe
*T_z_*	temperature of the inner wall of the outer pipe
*U*	voltage
**Subscripts**	
*a*	ambient
*l*	liquid
*p*	PCM
*s*	solid
*t*	tube
*w*	water
0	initial value
1	before phase transition
2	during phase change
3	after phase transition
